# Children's Behavior and Maternal Parenting Stress in Young Children With Sex Chromosome Trisomies

**DOI:** 10.1097/DBP.0000000000001015

**Published:** 2021-10-11

**Authors:** Alessandra Lorini, Laura Zampini, Gaia Silibello, Francesca Dall'Ara, Claudia Rigamonti, Paola Francesca Ajmone, Federico Monti, Faustina Lalatta, Maria Antonella Costantino, Paola Giovanna Vizziello

**Affiliations:** * Department of Psychology, University of Milano-Bicocca, Milan, Italy;; † Child and Adolescent Neuropsychiatric Unit, Foundation IRCCS Ca' Granda Ospedale Maggiore Policlinico, Milan, Italy;; ‡ Clinical Genetics Unit, Foundation IRCCS Ca' Granda Ospedale Maggiore Policlinico, Milan, Italy.

**Keywords:** sex chromosome trisomies, behavioral problems, maternal parenting stress, psychomotor development, language development

## Abstract

**Objective:**

Children and adolescents with sex chromosome trisomies (SCTs) usually show a higher frequency of behavioral problems than typically developing (TD) children. However, little is known about the presence of behavioral issues in toddlers with SCT. This study aimed at investigating their behavioral profile in the second year of life and its impact on maternal stress.

**Method:**

Participants were 87 children ranging in age from 18 to 26 months: 63 children with SCTs (all diagnosed prenatally) and 24 TD children. Their psychomotor and language development and their behavioral profile were assessed. In addition, the level of maternal parenting stress was evaluated*.*

**Results:**

Both psychomotor and language development were significantly lower in children with SCTs than in TD children. Conversely, no significantly greater behavioral problems emerged in children with SCTs. However, a significantly higher level of parenting stress related to a dysfunctional interaction with the child emerged in the mothers of children with SCTs. In this population, maternal stress seemed positively related to their children's emotional problems and pervasive disorders and negatively related to their children's psychomotor and linguistic competence.

**Conclusion:**

Although no significant behavioral issues emerged in the second year of life, the relationships found between children's behavioral profiles and maternal parenting stress highlight the importance of prenatal counseling and support groups for parents of children with SCTs. This might help them recognize the first signs of behavioral problems and become aware of their influence on parenting stress.

Sex chromosome trisomies (SCTs) are genetic conditions characterized by 1 more sex chromosome than the 2 in typical karyotypes. In the female population, the additional chromosome is an X chromosome, and the condition is known as triple X syndrome (XXX syndrome), whereas in the male population, it could be either X (Klinefelter syndrome or XXY syndrome) or Y (Jacobs syndrome or XYY syndrome). These syndromes have a prevalence ranging from 1/650 to 1/1000 born babies, and Klinefelter syndrome seems to be the most frequent.^1^

Children with SCTs usually have intelligence in the normal range, although they score on average about 10 points less than typically developing (TD) children in standardized tests.^2,3^ There is almost always a discrepancy between verbal and performance intelligence quotients in individuals with XXY syndrome, with the former being the most affected.^2^ However, this discrepancy is not always found in studies on people with XYY syndrome and XXX syndrome.^4^

Children with SCTs frequently show deficits in executive functions.^5^ For instance, children with an extra X (i.e., female children with XXX syndrome and male children with XXY syndrome) usually show higher difficulties in inhibition, mental flexibility, sustained attention, and working memory than TD children.^6^ Conversely, Janusz et al.^7^ showed that attention is the most affected executive function domain in children with XXY syndrome, whereas inhibition is relatively preserved. Concerning XYY syndrome, Ross et al.^8^ found that all males with SCTs, ranging in age from 4 to 14 years, show deficits in executive functioning tasks, but children with XYY syndrome show more cognitive flexibility problems than children with XXY syndrome. Some cognitive function deficits are also found in adults with XXY syndrome, especially in cognitive flexibility, planning, inhibition, and working memory.^9^

Children with SCTs frequently show delays and impairments in language development compared with TD children.^10^ These differences are detectable from the early stages of language development,^11,12^ and the proportion of children with SCTs who received a diagnosis of language impairment is significantly higher than in the general population.^10^ Moreover, children with SCTs frequently show learning impairments at school age because of their language problems.^2^

People with SCTs also show a higher incidence of behavioral problems than the general population, including attention-deficit/hyperactivity disorder (ADHD),^3^ autism spectrum disorder (ASD),^5,13^ anxiety and depression,^5^ and social withdrawal.^14^ Urbanus et al.^15^ highlighted how behavioral problems in children with SCTs are often present but more blurred in the early years of life because the impact of behavioral problems generally tends to grow in late toddlerhood and preschool age. In their recent study, Urbanus et al.^15^ assessed 87 children with SCTs, some ascertained by prenatal diagnosis and others diagnosed because of medical problems and developmental delays. The children's behavior and emotional development were assessed by parental reports. The authors found that socioemotional difficulties, behavioral problems related to affectivity, and pervasive developmental disorder begin to appear in the group of children ranging in age from 24 to 47 months rather than in the group of children ranging in age from 11 to 23 months.

As Geschwind and Dykens^16^ hypothesized, behavioral problems in the population of SCTs seem to be related to the type of extra chromosome: The presence of an extra X chromosome has been associated with a higher probability to show internalizing problems, whereas the presence of an extra Y chromosome has been associated with a higher probability to show externalizing problems. In children with XXY and XXX syndromes, internalizing behaviors such as low self-esteem, shyness, anxiety, and social withdrawal are frequently detected.^14^ Conversely, children with XYY showed more impulsiveness and hyperactivity symptoms, although inattention symptoms are commonly detected in all the SCTs, and the prevalence of ADHD is 3%–10% higher in children with SCTs than in the population of TD children.^3^

Concerning internalizing traits, issues such as anxiety and depression, social withdrawal, and even autistic traits have been observed in children with SCTs.^5^ Anxiety and depression seem more present in children with an extra X, but not in children with an extra Y in the karyotype.^2,17^ In XXY syndrome, 24% of male children and adolescents between age 6 and 19 years (12 cases of 51 participants) are diagnosed with depression, whereas 18% (9 cases of 51 participants) show anxious symptoms.^18^ In female children and adolescents with XXX syndrome (evaluated from 6 months to 24 years), depression is present in 18% of the cases (8 cases of 45 participants), whereas anxiety is present in 20% (9 cases of 45 participants).^19^ In male children with XYY, no higher rates of anxiety and depression using the Child Behavior Checklist^20^ have been observed compared with TD children.^17^ Social withdrawal is often present in children with XXY syndrome,^21^ and it remains stable even when they reach adulthood.^22^ Van Rijn et al.^23^ have observed how female children with XXX syndrome's social difficulties are similar to those of male children with XXY syndrome. Conversely, shyness and social withdrawal seem less frequent in male children with XYY syndrome than in children with an extra X in the karyotype.^24^ This result is consistent with the study by Geschwind and Dykens,^16^ which emphasizes the presence of major internalizing problems, such as withdrawal, in children with an extra X. Another internalizing problem that could be found in children with SCTs is the presence of autistic traits.^5^ These are observed in 35% of children with XYY syndrome^25^ and 19% of children with an additional X chromosome.^23^ The diagnosis rate of ASD is higher in children with SCTs than in TD children: 19% of male children with XYY syndrome and 11% of male children with XXY syndrome.^13^ In female children with XXX syndrome, the situation is less clear: In the study of Bishop et al.,^13^ no diagnosis of ASD was found in female children, whereas in the study of Van Rijn et al.,^5^ 15% of female children with XXX syndrome received a diagnosis of ASD or showed significant clinical symptoms.

Concerning externalizing traits, children with SCTs frequently show problems related to both attention and aggressive behavior, which are also present in children with ADHD.^5^ Tartaglia et al.^3^ pointed out that symptoms related to ADHD are present in SCTs at 3%–10% higher rates than in the general population. in all of the three types of SCTs, inattention-related symptoms are frequently detected, whereas children with XYY syndrome also show more impulsiveness and hyperactivity.^3^ Externalizing behaviors, including aggressive behavior and attention problems, are more frequent in this syndrome than in the general population and the other SCTs.^16^ The cause of these aggressive behaviors is neither clear nor univocal: The deficit of executive functions could be influential but also the impulsive symptoms related to ADHD typical of this syndrome.^4^ In fact, deficits in executive functions, particularly inhibition problems, may be associated with more externalizing behaviors,^6^ symptoms of ADHD, and social difficulties.^26^

## Aims of the Study

Children and adolescents with SCTs usually show a higher incidence of internalizing and externalizing behaviors than TD children. However, to date, there is very little knowledge about the behavioral profile of young children with SCTs. This study aimed to analyze behavioral problems in this population during the second year of life, considering only children diagnosed through prenatal screening. In the prevention perspective, early identification of any eventual fragility allows intervening before the problems have stabilized. Moreover, we aimed at analyzing the impact of any possible internalizing or externalizing emerging issues on maternal parenting stress.

## METHODS

### Participants

Participants included in this study were 87 children ranging in age from 18 to 26 months (M = 23.59; SD = 2.18). Among these, 63 (27 female children) were children with sex chromosome trisomies (SCTs), recruited through the Child and Adolescent Neuropsychiatric Unit of the Foundation Istituto di Ricovero e Cura a Carattere Scientifico (IRCCS) Ca' Granda Ospedale Maggiore Policlinico, a public hospital in Milan (Italy), and 24 (11 female children) were typically developing (TD) children who were participating in a research project on language development at the Department of Psychology of the University of Milano-Bicocca (Milan, Italy). All children with SCTs were diagnosed prenatally by amniocentesis or chorionic villus: 27 children were diagnosed with XXX syndrome, 22 children with XXY syndrome, and 14 children with XYY syndrome. The parents of these children were recruited among those who received a prenatal diagnosis of an SCT at the Clinical Genetics Unit of the hospital between 2013 and 2019. All the parents were asked to participate in a monitoring program for children with SCTs. The response rate was 92%. The parents of TD children were recruited through a written invitation, which was sent to parents based on birth records provided by the municipality of Milan (Italy). None of the participants, either with SCTs or TD, showed additional neurological or genetic conditions. They all had normal hearing and no history of ear infections, and they all belong to monolingual Italian-speaking families. All parents were White, and their demographic information, such as age, level of education, and the number of children, is presented in Table 1. Both fathers and mothers were significantly older in the SCT group. This difference could be explained considering that mothers who chose to undergo prenatal screening are usually older than those who do not, and all the children in the SCT group were diagnosed before birth. In addition, both fathers and mothers had a significantly higher education level in the TD group. This difference could be explained considering that children in the TD group were recruited on a voluntary basis to participate in a university research project. Conversely, children in the SCT group came from a more heterogeneous group of families participating in a hospital monitoring program. This is also the reason for the different number of participants in the 2 groups. No significant differences were found in the number of children per family. Children's parents signed a written informed consent form before inclusion in this study.

**Table 1.** Participant's Demographic Characteristics

	SCT Group		TD Group		*F*_*(1,83)*_	*p*	*η* ^*2*^
	M	SD	M	SD			
Father's age	41.93	4.22	37.04	6.51	16.74	<0.001	0.17
Father's year of education^a^	13.82	3.67	16.25	2.80	8.55	0.004	0.09
Mother's age	40.00	4.12	35.38	4.95	19.33	<0.001	0.19
Mother's year of education^a^	14.62	3.46	16.83	1.90	8.73	0.004	0.10
No. of children	1.48	0.65	1.29	0.62	1.41	0.238	0.02

a In the Italian education system: primary school license (5 yr), middle school license (8 yr), high school diploma (13 yr), bachelor degree (16 yr), master degree (18 yr).

SCT, sex chromosome trisomies; TD, typically developing.

Children with SCTs were assessed on average at 23.84 months (SD = 2.03), whereas children in the TD group were assessed on average at 22.92 months (SD = 2.45). An analysis of variance confirmed no significant differences in the age at which the children were assessed (*F*_(1,85)_ = 3.20; *p* = 0.077).

### Procedure

The children behavior and linguistic skills were assessed indirectly through parental reports. Mothers filled out the Italian version of the Child Behavior Checklist (CBCL)^27^ and the Italian version of the MacArthur-Bates Communicative Development Inventories-Words and Gestures Form (Il Primo Vocabolario del Bambino [PVB]^28^).

The CBCL aimed to investigate the presence of possible behavioral problems in children. The CBCL 1.5-5 years version was used in this study because of the participant's age range. The CBCL 1.5-5 is a parental questionnaire consisting of 100 statements concerning child behavior. The parents are asked to state on a 3-point Likert scale whether each statement is “not true” (0), “somewhat or sometimes true” (1), or “very true or often true” (2) looking back on the past 2 months. The scores can be grouped into 7 cross-informant syndrome scales: emotional reactions, anxiety/depression, somatic complaints, withdrawal, problems of sleep, attention problems, and aggressive behaviors. These scales could be then grouped into 2 principal scores: internalizing problems (obtained from the sum of the scores in the scales emotional reactions, anxiety/depression, somatic complaints, and withdrawal) and externalizing problems (obtained from the sum of the scores in the scales attention problems and aggressive behaviors). In addition, from the CBCL scores, it is also possible to compute the scores in 5 Diagnostic and Statistical Manual of Mental Disorders (DSM)-oriented scales that are structured in a way compatible with the diagnostic categories of the DSM classification: Emotional Problems, Anxiety Problems, Pervasive Developmental Disorder, Attention-Deficit/Hyperactivity Disorder, and Oppositional Defiant Disorder. Similar to recent studies on this topic,^15^ we decided to consider these latest scores. The scores of the 5 DSM-oriented scales were then converted into T scores (M = 50; SD = 10). T scores above 70 are considered in the clinical range, and the scores between 65 and 70 are in the borderline range.

The PVB aimed to assess the children's vocabulary size. The mothers were asked to sign, on a checklist of approximately 700 words, the words that their children could produce spontaneously. Raw scores were considered in this study.

To evaluate the level of maternal parenting stress, we used the Parenting Stress Index–Short Form [PSI].^29^ The PSI is a Likert-type parental self-assessment questionnaire designed to measure stress in the parent-child system. The questionnaire consists of 36 items divided into 3 main subscales: (1) Parental Distress (e.g., stress deriving from parents' perception of their child-rearing competence and stress deriving from restrictions on their social life), (2) Parent-Child Dysfunctional Interaction (e.g., stress deriving from interactions that do not meet parents' expectations), and (3) Difficult Child (e.g., stress deriving from parents' view of the child temperament or noncompliance). Each item rates from 1 (strongly disagree) to 5 (strongly agree). Raw scores are converted in percentiles. High scores indicate a higher level of stress. Parents scoring above the 85th percentile are considered to be experiencing clinical levels of parenting stress.

Besides the indirect evaluation through parental reports, the children's psychomotor development was directly assessed by administering the Griffiths Mental Development Scales [GMDS].^30^ The GMDS allow computing a general developmental quotient (DQ) concerning 5 different domains: Locomotor, Personal-Social, Language, Eye and Hand Coordination, and Performance. The DQ has a distribution with M = 100 and SD = 15.

### Data Analysis

Data analysis was performed using IBM SPSS Statistics 26. First, univariate analysis of variance (ANOVA) was used to assess statistically significant differences in DQ and vocabulary size between the 2 groups of children (SCT group vs TD group). A multivariate ANOVA (MANOVA) was then performed, with group (SCT vs TD) as a between-subject factor, specifying the CBCL-DSM–oriented scales as dependent variables. Next, another MANOVA was performed to assess the between-group differences in maternal parenting stress. Finally, for the SCT group, a hierarchical regression analysis was performed to understand the contribution of children's behavior (CBCL-DSM–oriented scales) and children's cognitive (GMDS DQ) and linguistic (PVB vocabulary size) competence in explaining the individual variability in maternal stress. The level of significance was set at *p* ≤ 0.05 (2-tailed).

## RESULTS

### Cognitive and Linguistic Development

The 2 groups of children significantly differ in their developmental quotient (DQ), as assessed by the administration of the GMDS (*F*_(1,80)_ = 17.86; *p* < 0.001; *η*2 = 0.18). In particular, children in the sex chromosome trisomies (SCT) group showed a significantly lower DQ than the children in the typically developing (TD) group (SCT: M = 98.79; SD = 12.31. TD: M = 110.21; SD = 7.43), although in the normal range. Concerning children's linguistic development, as assessed by the administration of the Italian version of McArthur-Bates Communicative Development Inventory, the vocabulary size of children with SCTs was significantly lower than that of TD children (SCT: M = 73.43; SD = 93.61. TD: M = 221.83; SD = 207.33. *F*_[1,83]_ = 20.79; *p* < 0.001; *η*^*2*^ = 0.20).

### Behavioral Problems

Considering the Child Behavior Checklist (CBCL)-DSM–oriented scales, 6 in 63 children with SCTs (9.52%) and 1 in 24 TD children (4.17%) showed scores in the clinical (T scores above 70) or borderline (T scores between 65 and 70) range. In particular, in the SCT group, 1 child with XXX was in the clinical range for Pervasive Developmental Disorder (PDD), 3 children (2 with XXX and 1 with XXY) were in the borderline range for PDD, 1 female child with XXX was in the borderline range for Attention-Deficit/Hyperactivity Disorder, and 1 female child with XXX was in the borderline range for Oppositional Defiant Disorder. In the TD group, only 1 male child was in the borderline range for PDD. The children in the 2 groups did not show any significant differences in their mean scores at the CBCL-DSM–oriented scales (see Table 2).

**Table 2.** MANOVA of the CBCL-DSM–Oriented Scales

	SCT Group		TD Group		Univariate Analysis			Multivariate Analysis		
	M	SD	M	SD	*F*_*(5,82)*_	*p*	*Part η* ^*2*^	*F*_*(5,81)*_	*p*	*Part η* ^*2*^
								2.32	0.051	0.13
Emotional Problems	51.75	2.97	51.83	3.28	0.01	0.906	0.00			
Anxiety Problems	50.92	2.26	51.21	1.91	0.31	0.582	0.00			
Pervasive Developmental Disorder	53.73	5.46	52.04	3.69	1.94	0.167	0.02			
ADHD	51.92	3.40	54.04	6.76	3.76	0.056	0.04			
Oppositional Defiant Disorder	50.75	2.48	50.50	0.83	0.22	0.637	0.00			

ADHD, attention-deficit/hyperactivity disorder; CBCL, Child Behavior Checklist; MANOVA, multivariate analysis of variance; SCT, sex chromosome trisomies; TD, typically developing.

### Maternal Parenting Stress

Concerning parenting stress, 7 in 63 mothers in the SCT group (11.11%) and 1 in 22 mothers in the TD group (4.55%) showed scores in the clinical range. In particular, in the SCT group, the mothers of 4 female children with XXX and 3 male children with XXY showed clinical scores in at least one of the Parenting Stress Index–Short Form (PSI) subscales. In the TD group, the mother of a female child showed a clinical score in the PSI Difficult Child scale. The mothers in the 2 groups showed a statistically significant difference in the PSI Parent-Child Dysfunctional Interaction subscale scores, with the mothers of children with SCTs perceiving a higher level of stress than the mothers in the TD group. By contrast, no significant differences were found in the PSI Parenting Distress and the PSI Difficult Child scales (see Table 3). In both groups, maternal parenting stress was not significantly related to maternal age (all rs < 0.38; all *p* values > 0.077) or education level (all rs > −0.20; all *p* values > 0.315).

**Table 3.** MANOVA of the PSI Subscales

	SCT Group		TD Group		Univariate Analysis			Multivariate Analysis		
	M	SD	M	SD	*F*_*(3,81)*_	*P*	*Part η* ^*2*^	*F*_*(3,80)*_	*P*	*Part η* ^*2*^
								2.64	0.055	0.09
PSI Parenting Distress	33.87	26.52	28.27	23.11	0.77	0.382	0.01			
PSI Parent-Child Dysfunctional Interaction	38.56	22.41	26.41	16.27	5.44	0.022	0.06			
PSI Difficult Child	41.53	26.41	42.27	24.48	0.13	0.909	0.00			

MANOVA, multivariate analysis of variance; PSI, Parenting Stress Index–Short Form; SCT, sex chromosome trisomies; TD, typically developing.

### Predictors of Parenting Stress in Children With Sex Chromosome Trisomies

Since the maternal parenting stress related to the perception of a dysfunctional interaction was significantly higher in the SCT group, we computed the proportion of explained variance of this variable in the mothers of children with SCTs. We used a stepwise regression with 2 blocks of predictors. In the first one, we put the scores obtained by children in the CBCL-DSM–oriented scales, and in the second one, children's DQ and vocabulary size (Il Primo Vocabolario del Bambino). The results in Table 4 showed that the PSI Parent-Child Dysfunctional interaction subscale scores were significantly predicted by the scores in the PDD scale and children's GMDS DQ, accounting for 43% of the total variance. Therefore, the mothers of children with SCTs who showed more difficulties in pervasive developmental behaviors and had lower DQs showed a higher level of stress in the interaction with their children.

**Table 4.** Predictors of PSI Parent-Child Dysfunctional Interaction in Mothers of Children With SCTs

		*R* ^*2*^	*R*^*2*^ *adj*	*F*	*ΔR* ^*2*^	*Β*
Model 1		0.34	0.33	27.87_(1, 54)_***		
	Emotional Problems					0.10
	Anxiety Problems					−0.10
	Pervasive Developmental Disorder					0.58***
	ADHD					0.02
	Oppositional Defiant Disorder					0.06
Model 2		0.48	0.43	21.45_(2, 53)_***	0.11**	
	Emotional Problems					0.12
	Anxiety Problems					0.01
	Pervasive Developmental Disorder					0.45***
	ADHD					0.04
	Oppositional Defiant Disorder					0.04
	GMDS DQ					−0.36**
	PVB vocabulary size					−0.16

Note: **p < .01; ***p < .001

ADHD, attention-deficit/hyperactivity disorder; DQ, developmental quotient; PSI, Parenting Stress Index–Short Form; PVB, Il Primo Vocabolario del Bambino; SCT, sex chromosome trisomies

## DISCUSSION

This study aimed to analyze emerging behavioral problems in children with sex chromosome trisomies (SCTs) during their second year of life. Data derived from both direct and indirect assessments of a group of children with SCTs, all diagnosed through prenatal screening, were compared with typically developing (TD) peers.

First, we compared the psychomotor and language development of the 2 groups of children. We found that both children's general developmental quotient (DQ) and vocabulary size were significantly lower in children with SCTs than in TD children. In particular, the DQ of children with SCTs was, on average, 11 points lower than that of the children in the control group. This finding reflects those on DQ and intelligence quotients reported in the literature on younger^11^ and older children with SCTs.^2^ Concerning children's vocabulary size, the number of words produced by children with SCTs was on average more than 3 times lower than that produced by TD children. Even this finding is in concordance with previous studies on the emergence of language in this population.^12,13^

We detected possible behavioral problems in the children using the Child Behavior Checklist (CBCL), as performed in previous studies on this topic.^15^ As found by Urbanus et al.,^15^ most children with SCTs scored within the nonclinical range on the CBCL. However, scores in the clinical or borderline range were found in approximately 10% of the children with SCTs and approximately 4% of TD children. These problematic scores were found mainly in the CBCL-DSM–oriented scale of Pervasive Developmental Disorder (PDD). This result is partially in concordance with the result of Urbanus et al.,^15^ who found that children with SCTs aged 2–5 years showed more behavioral symptoms of affective and pervasive developmental problems than children without SCTs. However, it must be noted that the children in the 2 groups did not show any significant differences in their mean scores at the CBCL-DSM–oriented scales. A similar result was found by Urbanus et al.^15^ in their group of children ranging in age from 18 to 24 months.

Concerning parenting stress, although only a minority of mothers showed scores in the clinical range (i.e., approximately 11% in the SCT group and 5% in the TD group), we found a significantly higher level of parenting stress related to a dysfunctional interaction with the child in the mothers of children with SCTs. Moreover, we found that the stress perceived by mothers of children with SCTs was significantly predicted by the scores obtained by children in the PDD scales and children's DQ. Therefore, a higher level of pervasive developmental behaviors and a lower cognitive competence could elicit a higher stress level in mother-child interaction. These data suggest considering an adequate level of children's psychomotor development as a possible protective factor for parenting stress in mother-child interaction within the population of children with SCTs.

The strength of this study is that all the participants with SCTs were diagnosed before birth by prenatal screening. This is particularly important in interpreting our results because previous studies have consistently shown that children with a postnatal diagnosis often have more behavioral issues than both TD children and prenatally diagnosed children with SCTs.^13,15,17^

### Clinical Implications

From the prevention perspective, the early identification of any possible behavioral issue is fundamental to plan early and targeted interventions. However, the lack of statistically significant differences between the 2 groups of children in the Child Behavior Checklist (CBCL) scores should not be interpreted as the absence of a behavioral problem. At age 2 years, behavioral issues might not have become apparent yet. In fact, we know from the literature^16^ that older children with sex chromosome trisomies (SCTs) frequently show many issues in both internalizing and externalizing behaviors. In addition, Urbanus et al.^15^ showed that these problems could emerge as early as at the age of 2 years.

Although only a few children and mothers scored in the clinical or borderline range in both CBCL and Parenting Stress Index–Short Form in the second year of life, we found that even mild internalizing issues could significantly affect mother-child interaction. Therefore, it is fundamental to discuss with mothers of children with a prenatal diagnosis of SCTs both the possible emergence of behavioral problems in their children and the possible effects of these behavioral problems on their own psychological well-being.

The early participation in support groups for parents with a prenatal diagnosis of SCT might help recognize the early signals of behavioral issues in children and reflect on how parenting stress could be related to children's behavior. Considering the rapid change in behavior that can occur in the first 2 years of life, we suggest the opportunity for parents of children with SCTs to participate in support groups conducted by expert clinicians already in the first months of life to sustain the early parent-child relationship and prevent possible fragilities.

### Study Limitations and Future Directions

This study has the value of considering a large and homogeneous group of young children with SCTs, all identified on prenatal screening, which means it is relatively unbiased, compared with studies on cases identified during investigations for developmental problems. However, a limitation of this study is the small number of children in the control group and the differences found between groups for parental age and education level. This limitation may depend on the different participation motivations of parents in the 2 groups. Parents in the typically developing group voluntarily agreed to participate in a research project promoted by the university. By contrast, parents in the SCT group were involved in a clinical follow-up program at the hospital. Another important limitation of this study is the transversal design that does not allow us to analyze the changes over time in children's behavior. Future studies should address when and how internalizing and externalizing behaviors emerge in the population of children with SCTs. A longitudinal design should be useful to outline the developmental trajectories of children's behavioral issues and their relationships with maternal parenting stress.
